# Oral Health Status in Patients with Head and Neck Cancer before Radiotherapy: Baseline Description of an Observational Prospective Study

**DOI:** 10.3390/cancers14061411

**Published:** 2022-03-10

**Authors:** Cosimo Rupe, Alessia Basco, Anna Schiavelli, Alessandra Cassano, Francesco Micciche’, Jacopo Galli, Massimo Cordaro, Carlo Lajolo

**Affiliations:** 1 Head and Neck Department, Fondazione Policlinico Universitario A. Gemelli—IRCCS, School of Dentistry, Università Cattolica del Sacro Cuore, Largo A. Gemelli, 8, 00168 Rome, Italy; cosimorupe@gmail.com (C.R.); alessiabasco19@gmail.com (A.B.); massimo.cordaro@policlinicogemelli.it (M.C.); carlo.lajolo@policlinicogemelli.it (C.L.); 2 Department of Medical Oncology, Fondazione Policlinico Universitario A. Gemelli—IRCCS, Institute of Radiology, Università Cattolica del Sacro Cuore, Largo A. Gemelli, 8, 00168 Rome, Italy; alessandra.cassano@policlinicogemelli.it; 3 Department of Radiation Oncology, Fondazione Policlinico Universitario A. Gemelli—IRCCS, Institute of Radiology, Università Cattolica del Sacro Cuore, Largo A. Gemelli, 8, 00168 Rome, Italy; francesco.micciche@policlinicogemelli.it; 4 Head and Neck Department, Fondazione Policlinico Universitario A. Gemelli—IRCCS, Institute of Otolaryngology, Università Cattolica del Sacro Cuore, Largo A. Gemelli, 8, 00168 Rome, Italy; jacopo.galli@policlinicogemelli.it

**Keywords:** head and neck cancer, oral status, periodontitis, dental caries, DMFt

## Abstract

**Simple Summary:**

Patients with head and neck cancer (HNC) are often considered as a group with compromised oral conditions, but this idea is not sufficiently supported by data in the literature. This study examined the oral condition—specifically the presence of caries and periodontal disease—of a cohort of patients with HNC waiting to start radiation therapy treatment and possible correlations between oral health, different types of HNC and various risk factors. The results confirm that the oral status of many patients with HNC is poor even before radiotherapy treatments and that smoking habit and tumor site are associated with poor oral health. These findings underline the importance of a dentist within a head and neck tumor board (TB), so that oral health can be restored as soon as possible.

**Abstract:**

(1) Background: The general hypothesis that HNC patients show compromised oral health (OH) is generally accepted, but it is not evidence-based. The objective of this baseline report of a prospective observational study was to describe the oral health of a cohort of patients with HNC at the time of dental evaluation prior to radiotherapy (RT). (2) Materials and Methods: Two hundred and thirteen patients affected by HNC who had received an indication for RT were examined with the support of orthopantomography (OPT). The DMFt of all included subjects, their periodontal status and the grade of mouth opening were recorded. (3) Results: A total of 195 patients were ultimately included: 146/195 patients (74.9%) showed poor OH (defined as having a DMFt score ≥ 13 and severe periodontitis). The following clinical characteristics were correlated with poor oral health in the univariate analysis: tumor site, smoking habit and age of the patients (in decades); χ2 test, *p* < 0.05. (4) Conclusions: This study confirms that the OH of HNC patients is often compromised even before the beginning of cancer treatment and, consequently, highlights how important it is to promptly schedule a dental evaluation at the moment of diagnosis of the cancer.

## 1. Introduction

The head and neck region is an anatomical heterogeneous area that can give rise to a variety of malignancies and show different risk factors, prognoses and treatments. Head and neck cancers (HNCs) represent the seventh most common malignancy worldwide [1].

The general hypothesis that HNC patients show a high prevalence of caries and periodontitis and, therefore, compromised oral health (OH) even before cancer therapy (i.e., radiotherapy, RT) is generally accepted, but it is not evidence-based. In fact, it is possible to highlight a lack of clinical data about the OH of these patients before oncological treatments.

Several studies reported that the majority of HNC patients did not attend any dental visit during the year preceding the cancer diagnosis and that many of these patients consulted a dental specialist only in cases of acute pain or other urgencies [2,3,4]. The overlapping of some risk factors—the most important being smoking habit—might be another possible explanation for the compromised conditions of HNC patients. Tobacco smoking is considered the main risk factor for the majority of HNCs and one of the main risk factors for the onset and progression of periodontitis and for its response to treatment [5,6,7,8]; furthermore, hyposalivation following prolonged exposure to tobacco smoking could increase the risk of caries development [9,10].

Furthermore, especially when RT is performed, preserving OH becomes crucial in the multidisciplinary management of these patients, since RT increases the risk of developing dental caries, leading to tooth loss, a well-known risk factor for major complications such as osteoradionecrosis [11,12,13].

Considering this, it is easy to imagine that HNC patients have a higher probability of developing dental diseases. Nevertheless, data available from the literature are scarce, often inaccurate or incomplete, and many articles do not stratify the statistical analysis according to the primary location of the cancer. The present study is the first report of a prospective protocol aiming to evaluate the OH of an HNC cohort undergoing RT.

The primary objective of this cross-sectional study was to describe the OH conditions of a cohort of HNC patients evaluated during the dental visit preceding RT. The secondary objective was to identify a correlation between the clinical characteristics of the patients and their OH status.

## 2. Materials and Methods

This study was conducted according to the Declaration of Helsinki, and all patients signed an informed consent form. The protocol was approved by the Ethics Committee of the Università Cattolica del Sacro Cuore (Ref. 22858/18) and was registered at ClinicalTrials.gov (ID: NCT04009161).

Patients affected by HNC attending the Oral Medicine, Head and Neck Department —Fondazione Policlinico Universitario A. Gemelli—IRCSS, between March 2017 and September 2021 were consecutively recruited in this study.

The following inclusion criteria were considered: HNC diagnosis and indication for RT.

The exclusion criteria were the impossibility of accurately evaluating OH conditions (i.e., outcomes of oncologic surgery incompatible with the dental procedures to diagnose caries and periodontitis) and patients having already received RT in the head and neck region.

All patients were visited prior to RT, with the support of an orthopantomograph (OPT). Firstly, anagraphic and anamnestic data were carefully recorded, particularly focusing on the oncologic history of the patient and on exposure to risk factors for oncologic and dental diseases.

Subsequently, the clinical evaluation of the following parameters was performed: presence of dental caries and DMFt score, periodontal health, maximal mouth opening (MMO).

The DMFt index is the key measure of caries experience in dental epidemiology [14]. It sums the number of decayed teeth, missing teeth due to caries and filled teeth in the permanent dentition. An examination for dental caries in permanent teeth is performed, examining 32 teeth. The permanent dentition status of each tooth (crown and root) is recorded as a score, where 0 corresponds to a tooth that shows no evidence of treated or untreated caries, and 1 corresponds to the case of tooth decay (treated or untreated) or a missing tooth (due to caries) [15].

The diagnosis of caries was performed through the clinical examination with the help of a dental explorer and a mouth mirror and, when in doubt, with the support of an intraoral radiograph (periapical or bitewing), performed with the help of film holders (Dentsply Sirona, Rome, IT). A bitewing radiograph was performed in every case in which visual inspection of the interproximal tooth surface was not possible. Nevertheless, when a diagnosis of an endodontic or periodontal lesion had to be performed, a periapical radiograph was taken. Caries involving the dentine were considered in the DMFt score (ICDAS™ code 3 and higher) [16,17,18].

Clinical evaluation of periodontitis was performed according to international standards [19]. A full-mouth periodontal examination was performed by the same operator (L.C.), with more than ten years of experience in periodontology, using an NCP15 periodontal probe and collecting the following data (six sites for each tooth): periodontal probing depth (PPD), the distance between the tip of the periodontal probe and the gingival margin; gingival recession (REC), the distance between the gingival margin and the cementoenamel junction; clinical attachment loss (CAL) for each assessed site; furcation involvement (FI), according to the Hamp classification [20]; number of tooth losses due to periodontitis; tooth mobility; full-mouth plaque score (FMPS) [21]; and full-mouth bleeding score (FMBS) [22].

After data collection, the periodontal cases were staged according to the diagnostic criteria of the 2017 classification: CAL ≥ 2 mm affecting two nonadjacent teeth, buccal or oral CAL ≥ 3 mm and PPD > 3 mm affecting two or more teeth were the diagnostic criteria to define a periodontitis case. Interdental CAL from 3 to 4 mm was the parameter which shifted the diagnosis to stage II periodontitis, while more severe CAL or at least one tooth lost due to periodontitis was the criterion which determined the shift to stage III or IV periodontitis. The differential diagnosis between stage III and IV periodontitis was driven by the following parameters: tooth loss due to periodontitis ≥ 5, masticatory dysfunction due to secondary occlusal trauma, bite collapse, drifting or flaring, which were the diagnostic criteria for stage IV periodontitis [19]. The clinical charts of the patients visited before 2017 were rescreened to stage the periodontal cases according to the above-mentioned classification. OPT was used as a support to complete the diagnosis and staging of periodontitis; in case of uncertainty, an intraoral radiograph was performed, compatible with the outcomes of the major oncologic surgery.

The M parameter (teeth missed due to caries), as well as the number of teeth lost due to periodontitis, was evaluated by analysing old radiographic exams provided by the patients. In case old radiographic exams were unavailable, the patients were asked about the reason for previous teeth extractions.

The MMO was defined as the greatest distance (mm) between the incisal edge of the maxillary central incisor and the incisal edge of the mandibular central incisor and was measured by using a modified vernier caliper [23]. The MMO of the edentulous patients was measured by removing every removable prosthesis, and the edentulous ridges were used as reference points.

The following variables were recorded: sex, age, risk factors (smoking, diabetes), previous or scheduled oncological treatment (chemotherapy and surgery), site, histological type and stage of the tumor, DMFt, stage of periodontitis and MMO.

The oral health (OH) parameter was defined as a dichotomous variable, and DMFt and periodontal staging were used to define OH status, defined as “poor” in cases of DMFt ≥ 13 and/or stage III or IV periodontitis and as “good” only in cases of lower values of each of these variables. The DMFt score of 13 was chosen as a cut-off defining good OH, since it has been reported to be the mean value of DMFt in non-developing countries [24,25]. Stage III and IV periodontitis were chosen as cut-off values, since they define “severe” periodontitis, according to the 2017 classification [19].

STROBE guidelines were followed to write this paper (Table S1).

### Statistical Analysis

The sample size was calculated according to the simple causal sampling formula. Considering a DMFt ≥ 13 and/or stage III or IV periodontitis as predictive of poor OH, and setting the possible prevalence of poor OH at 85% and a desired precision of 5%, 195 patients were included in the final sample.

Qualitative variables were described using absolute and percent frequencies, whereas quantitative variables were summarized either as the mean and standard deviation (SD), if normally distributed, or as the median, otherwise.

The following variables were evaluated as absolute values and reclassified in ranges. DMFt was reclassified according to the established cut-off defining a poor OH condition: DMFt ≥13; periodontitis was reclassified into three categories: absence of periodontitis, stage I or II periodontitis and stage III or IV periodontitis; MMO was reclassified according to the reduced mouth opening cut-off: MMO ≤ 25 mm [26,27]. DMFt and periodontal staging were used to define OH status as either “poor” or “good”, as described in the Materials and Methods section.

Correlation analysis between the OH parameters (DMFt and periodontitis) and the clinical characteristics of the patients was performed. The Kolmogorov–Smirnov test was performed to evaluate the normal distribution of the quantitative variables. The Mann–Whitney U test and Kruskal–Wallis test were performed to compare continuous variables with nonparametric distributions, whereas parametric variables were analyzed using ANOVA. Pearson’s χ2 test and Fisher’s exact test were used to compare discontinuous variables. A logistic regression model was built to evaluate factors affecting the probability of the main outcome variable (“poor OH”).

The statistical analysis was stratified according to the following variables: tumor site; patient age (by decade); and smoking habit.

Univariate analysis was performed to determine risk factors associated with poor OH (as defined in the Materials and Methods section), and the risk factors were introduced in a stepwise logistic regression analysis to identify independent predictors of poor OH. All statistical analyses were performed using IBM SPSS Statistics software (IBM Corp. Released 2017. IBM SPSS Statistics for Apple, Version 25.0 Armonk, NY, USA: IBM Corp).

## 3. Results

### 3.1. General Characteristics of the Population

Two hundred and thirteen patients were consecutively assessed and enrolled, while eighteen patients were excluded, since they did not fulfil the inclusion criteria (their clinical conditions did not allow clinical evaluation). The final sample included 195 patients (67 female and 128 male subjects), with a mean age of 60.4 years (SD: 12.4; range: 22–92). The mean time between the cancer diagnosis and the dental evaluation was 37.2 days (SD: 12.02; range: 15–64).

The general characteristics of the population are presented in Table 1. It is worth mentioning that the studied population represents a sample of a HNC population, reflecting the heterogeneous characteristics and risk factors for each malignancy.

#### 3.1.1. Oral Health

The clinical and radiographic evaluation showed that 8/195 (4.1%) subjects were totally edentulous, 115/195 (59%) showed a DMFt score ≥ of 13 and 150/195 (76.9%) were affected by periodontitis. Among these 150 patients, 107 (71.3%) showed stage III or IV periodontitis. Only 3/195 patients had a DMFt score = 0 (1.53%), while the median DMFt score was 16.91 (range: 0–32; SD: 9.1). A total of 146 patients out of 195 (74.9%) showed poor OH. The results describing the oral health of the studied population are reported in Table 2.

#### 3.1.2. Tumor Localization and OH Conditions

Patients with different tumor sites showed different OH conditions (χ2 test, *p* <0.05), with the larynx being associated with poor OH (86.4% of the cases) and the rhinopharynx being associated with good OH conditions (56.5%). The prevalence of DMFt ≥ 13 was higher in salivary gland (80%) and laryngeal (75%) patients than in patients with other tumor sites (χ2 test, *p* < 0.05). The subjects affected by laryngeal tumors also had a high prevalence of stage III or IV periodontitis, although this association was not statistically significant. The results of the statistical analysis, stratified according to the localization of the tumor, are reported in Table 3.

#### 3.1.3. Smoking and OH Conditions

**Figure 1:**
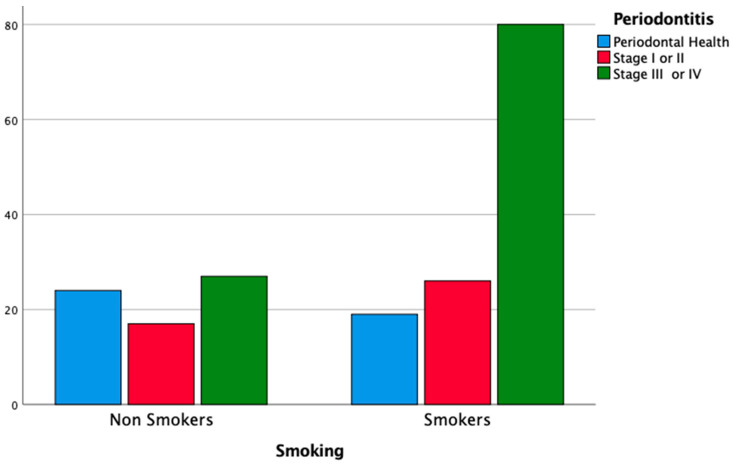
Distribution of periodontitis according to the habit of smoking.

**Figure 2:**
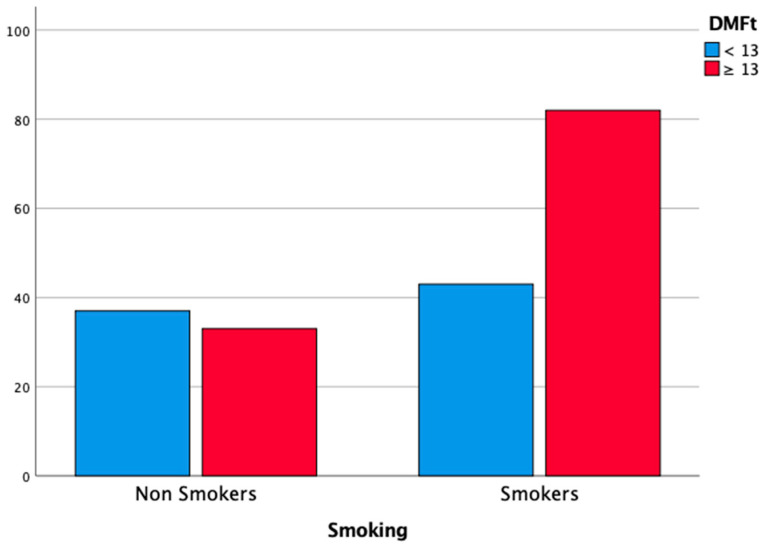
Distribution of DMFt < 13 according to the habit of smoking.

**Figure 3:**
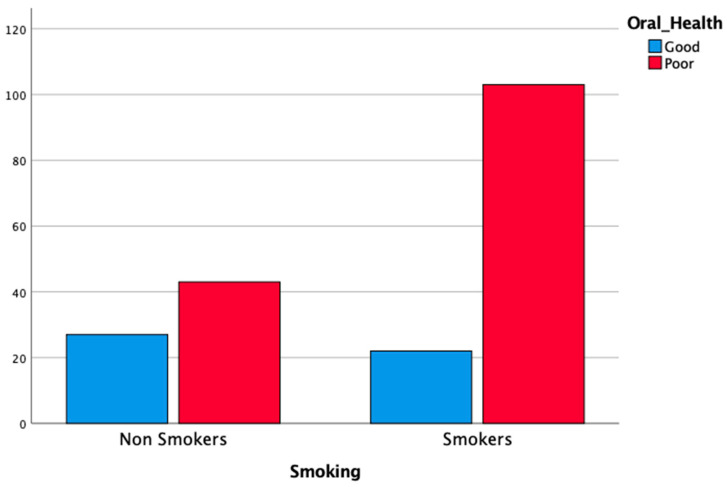
Distribution OH status according to the habit of smoking.

Smoking habit was correlated with the diagnosis of periodontitis: 74.8% of severe periodontal patients (stage III or IV) were smokers or former smokers (χ2 test, *p* < 0.05). The habit of smoking was also correlated with DMFt ≥ 13 (71.3%; χ2 test, *p* < 0.05) and poor OH (70.5%; χ2 test, *p* < 0.05). Multiple logistic regression analysis confirmed that smoking habit was a risk factor for severe periodontitis (OR = 4.78; 95% CI = 2.01–11.36; *p* < 0.05), for DMFt ≥ 13 (OR = 2.30; 95% CI = 1.19–4.44; *p* < 0.05) and, therefore, for poor OH (OR = 3.27; 95% CI = 1.46–7.33; *p* < 0.05). The results of the analysis, stratified according to smoking habit, are reported in Table 4, and Figure 1, Figure 2 and Figure 3.

#### 3.1.4. Age and OH Conditions

**Figure 4:**
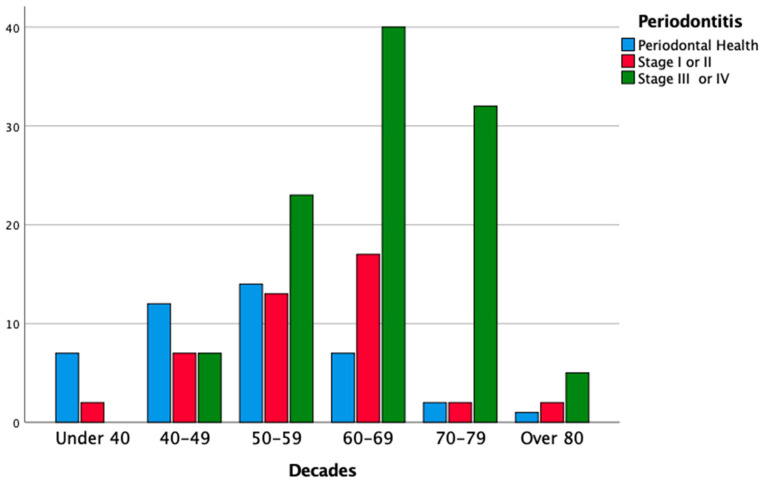
Distribution of periodontitis according to age of the patients (in decades).

**Figure 5:**
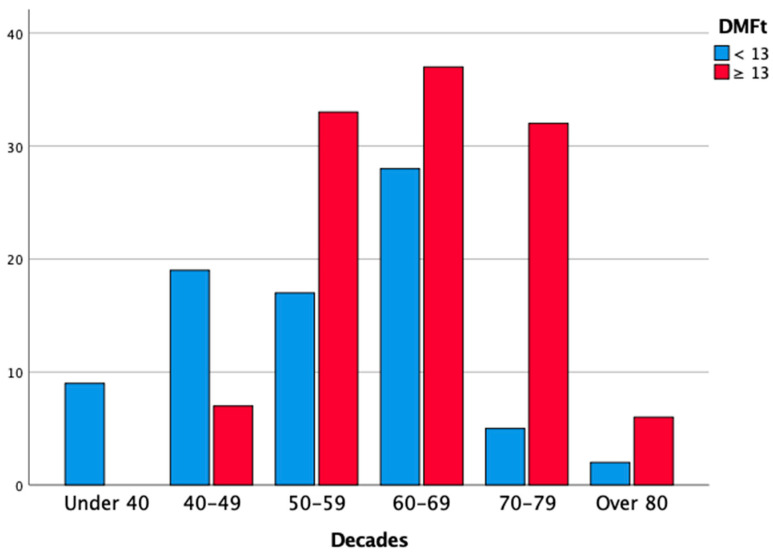
Distribution of DMFt < 13 according to age of the patients (in decades).

**Figure 6:**
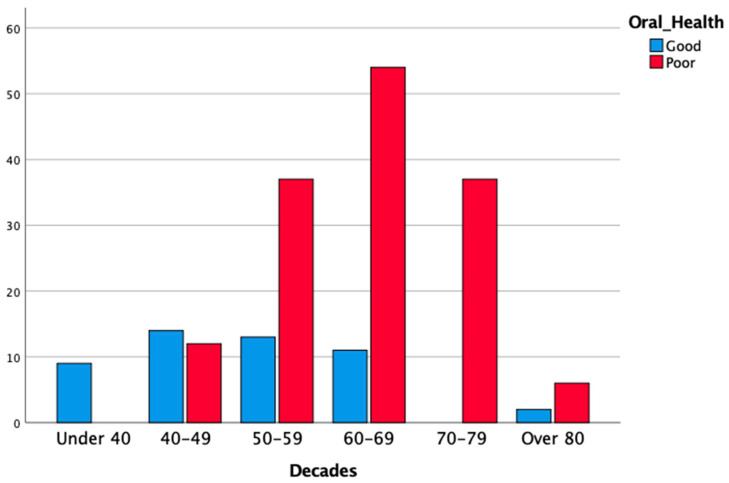
Distribution of OH status according to age of the patients (in decades).

The cases of severe periodontitis (stages III and IV) were diagnosed only in subjects aged > 40 years, and 93.5% of periodontal patients were older than 49 years (χ2 test, *p* < 0.05). Additionally, the distribution of high scores of DMFt (13 or higher) was not homogeneous (χ2 test, *p* < 0.05): DMFt scores of ≥ 13 were only found among subjects aged > 40 years, with a peak in the 70–79 years decade (86.4% of the subjects who were allocated to this decade) and in the > 80 years category (75%). Consequently, poor OH conditions were more prevalent among the elderly population, with a peak in subjects aged > 70 years (95.6% of subjects being older than 70 years). All edentulous patients in the studied population were older than 60 years (χ2 test, *p* < 0.05). Multiple logistic regression analysis showed that age (in decades) was a risk factor for periodontitis (stage I and II periodontitis: OR 1.73, 95% CI = 1.15–2.61; stage III and IV periodontitis: OR 3.30, 95% CI = 2.17–5.00; *p* < 0.05); for DMFt ≥ 13 (OR = 2.07; 95% CI = 1.53–2.79; *p* < 0.05); and for poor OH (OR = 2.98; 95% CI = 2.01–4.41; *p* < 0.05).The results of the analysis, stratified according to age, are reported in Table 5, and Figure 4, Figure 5 and Figure 6.

## 4. Discussion

The role of the dentist in the head and neck tumor board (TB) is becoming increasingly important, especially in the context of modern multidisciplinary management, which places greater emphasis on the quality of life of patients after, or during, cancer therapy. The results of the present work confirm the importance of a dental evaluation prior to RT to prepare a patient for these complex therapies.

Available studies regarding the oral status of subjects with HNC at the time of diagnosis are few and often inaccurate or incomplete [28]. From this lack and from the clinical impressions of many specialists derives the probably correct belief that HNC patients present poor OH. This idea is even more ingrained when it comes to subjects with oral cavity tumors.

The description of the oral status of the cohort of patients with HNC proposed by this study confirms, within the limits of a cross-sectional study, the generally accepted idea that subjects with HNC very often present poor OH, although this is not supported by the current literature.

In particular, the subjects of this cohort presented poor OH (DMFt ≥ 13 and/or periodontitis stage III or IV) in 74.9% of cases. The OH conditions were not equally distributed among the different tumor sites (χ2 test, *p* < 0.05): the subjects affected by SCC of the larynx (86.4%), of the salivary glands (86.6%) and of the oral cavity (78%) presented a higher prevalence of poor OH, when compared to the subjects affected by nasopharyngeal cancer (56.5% of nasopharyngeal patients presented DMFt < 13 and absence of severe periodontitis). Nevertheless, in the multivariate analysis, none of the tumor sites were revealed as an independent risk factor for poor OH.

The present work confirms that OH was more compromised the older the subjects were, with a peak (95.6% of cases) at 70 years of age and older (OR = 2.98; 95% CI = 2.01–4.41; *p* < 0.05). Multiple logistic regression analysis also showed that age was an independent risk factor for periodontitis (stage I and II periodontitis: OR 1.73, 95% CI = 1.15–2.61; stage III and IV periodontitis: OR 3.30, 95% CI = 2.17–5.00; *p* < 0.05) and for DMFt ≥ 13 (OR = 2.07; 95% CI = 1.53–2.79; *p* < 0.05).

The median DMFt value of the cohort analysed was 16.9. Fifty-nine percent of the included patients (115/195) had DMFt ≥ 13. Within the total population, only three subjects had DMFt = 0.

Although it is not possible to compare our results with those of previous works that studied cohorts of HNC patients, mainly due to the heterogenous methodology, some studies that reached similar conclusions can be found, such as those by Critchlow et al., Raskin et al. and Patel et al., who reported mean DMFt values of 19.6, 17.6 and 16.2 in HNC cohorts, respectively [28,29,30]. On the other hand, other studies (i.e., Jham et al. [31], Tezal et al. [32], Moraes et al. [33] and Kim et al. [34]) reported no significant correlation between HNC cancer and caries experience. Likely, the heterogeneity of the data stems from the different study designs, the criteria used in the different evaluations and the differences among the studied populations (i.e., geographical area, oral hygiene, access to dental care, distribution of different HNCs).

The percentage of subjects with periodontitis included in the present study was high (76.9%, 150/195) compared with epidemiological studies conducted in Europe, in which the prevalence of periodontitis did not exceed 70%, even in older age groups [35].

The classification of periodontitis proposed in 2017 [19] aims to remedy many of the critical issues present in epidemiological studies and to provide a more complete and detailed description of the populations under study. For this reason, in the present work, we chose to classify all cases of periodontitis based on this classification. In fact, if this study had limited itself to adopting the criteria proposed by previous classifications, many of the subjects with poor oral conditions, or a terminal dentition, would not have been included among the cases of severe periodontitis. The new classification, moreover, has made it possible to evaluate the periodontal status with a system based on two parameters (staging and grading) that, combined, provide information on the prognosis of the teeth and the complexity of the treatments required by the individual case. The combination of all this information constitutes a fundamental aid in deciding whether or not to perform extractions before RT.

Studies adopting the criteria proposed by the 2017 classification are very few [36,37,38], and our present work is the first to use them in a cohort of patients with HNC. Studies that have attempted to investigate a possible correlation between periodontitis and HNC are extremely diverse and often methodologically weak, as highlighted by a recent review [39]. In particular, the majority of studies did not adopt sound criteria to diagnose periodontitis [28,40,41,42,43,44,45,46,47,48,49,50], and only one [33] was based on a clinical evaluation integrated by the collection of truly suitable parameters (PPD and CAL).

Almost all authors who have analysed the OH of HNC patients before RT reported a high prevalence of periodontitis: Bonan et al. [51] reported a 93% prevalence of moderate or severe periodontitis, although cases were evaluated on the basis of a different classification; Moraes et al. [33] found that 80% of patients with oral and oropharyngeal SCC had generalized chronic periodontitis, almost exclusively severe. Although the results reported in the present study cannot be significantly compared with those of previous works because of methodological differences, they confirm that periodontitis, due to still unproven causes, is very common among patients with HNC.

Among the most plausible causes, the high incidence of smokers in these populations could play a key role. The data reported in our present study support this hypothesis; in fact, smokers represented 74.7% of the patients affected by stage III–IV periodontitis (80/107) (OR = 4.78; 95% CI = 2.01–11.36; *p* < 0.05). Statistical analysis showed that smoking also affected caries susceptibility (OR = 2.30; 95% CI = 1.19–4.44; *p* < 0.05) and, consequently, overall OH (OR = 3.27; 95% CI = 1.46–7.33; *p* < 0.05).

Despite the high percentage of patients with poor OH, only 8/195 (4.1%) were completely edentulous. The difference between the data reported by the present work and those of previous studies [4,31,51] may be influenced by the lower proportion of older individuals included in the present study (only 23.1% of patients were >70 years of age).

Interestingly, reduced MMO (<25 mm) did not correlate with the parameters of OH assessment. Reduced MMO is a very frequent clinical finding in HNC cohorts, as it can occur following both oncologic surgery and RT and makes dental care and inspection of the oral cavity particularly difficult, including during cancer follow-up appointments. However, a prospective study aiming to evaluate the correlation between MMO and OH is needed. It is very likely, in fact, that the greater difficulty in oral hygiene procedures, as well as in routine dental therapies and inspection procedures, due to a reduced MMO leads to an increase in the incidence of caries and a worsening of periodontal conditions.

This cross-sectional study has several strengths. The description of the oral status of the cohort is based on validated diagnostic and prognostic criteria, obtained through clinical and radiographic evaluation. Additionally, the reported results open the way to further investigating possible correlations between OH and HNC.

This study does not solely report the prevalence of caries and periodontitis in the analysed population; it proposes, for the first time, a criterion that may allow evaluating the OH of examined patients in a global and objective way. Establishing a cut-off to divide subjects into two groups according to the OH found emphasizes how defining an oral condition as “good” or “poor” is necessary, not only to find the presence or absence of caries and/or periodontitis, but also to quantify severity and to evaluate the two diseases through an integrated system.

Presenting a representative sample from each subsite of HNCs is one of the strengths of this study, as it provides a more specific picture of the OH conditions of patients with different HNCs. However, this also implies a limitation: the analysed sample, including subjects with tumors differing profoundly in risk factors and clinical manifestations, might be inhomogeneous. However, statistical analysis stratified by tumor subsites effectively allows highlighting the different peculiarities of individual HNCs from an OH perspective.

This study also has several limitations, among which, like all studies having evaluated the OH of HNC patients using DMFt as a parameter, is the retrospective attribution of the M parameter. This consideration also applies to the retrospective attribution of the number of teeth lost due to periodontitis. This necessity could lead to overestimating the prevalence of one pathology over another. However, the use of the “OH” parameter allows us to curb the extent of this potential bias, since it integrates the two main variables of interest.

In addition, a possible bias for this study is the lack of a control group, homogeneous to the one studied in terms of age, gender and smoking habits. More studies, with a different design (i.e., case–control studies), are needed to confirm that HNC patients have poorer OH than the general population.

Another parameter rendering the characteristics of the population peculiar is that all included patients had received an indication to undergo RT, since a dental visit is overwhelmingly indicated to prevent unwanted effects of RT. With this study being a real-life monocentric experience, indication for RT was chosen since the treatment of RT patients is the most “demanding”, both from oncological and dental points of view. Nevertheless, our study also includes patients that underwent major oncological surgeries. Their inclusion within our sample could have made the observed population more homogeneous in terms of OH variables, making our sample more representative of HNC patients than a population undergoing exclusive RT.

Nevertheless, it could be considered as a selection bias, since patients who underwent a major oncologic surgery often present poorer OH, due to the reduced ability to adequately perform oral hygiene procedures, resulting from surgical procedure-induced anatomic alterations. Nevertheless, the results of our study show that previous oncologic treatment did not have a statistically significant correlation with OH, somehow confirming that this possible bias did not have a great impact. This could be explained by the fact that the dental evaluation was carried out in a time-lapse not exceeding 60 days, an insufficient time frame to significantly influence the parameters analyzed in this study. Notwithstanding, the results of the present work demonstrate how HNC patients present poor OH even prior to RT, which makes their inclusion within a protocol of primary and secondary dental prevention indicated.

## 5. Conclusions

This work highlights, with a high level of evidence, the number of HNC patients presenting poor OH in the months immediately following their malignancy diagnosis and their consequent need for prevention protocols and highly rigorous dental therapy, considering the increasing number of patients undergoing RT.

With the time window between the dental evaluation and the start of RT being particularly narrow, performing multiple extractions becomes necessary, resulting in further worsening of the periodontitis stage and masticatory function. This can only be avoided by referring the patient to a dental team, who will commence necessary therapies and preventive measures. Moreover, due to the increasing rate of recurrences and second primary tumors, an increasing number of patients receive an indication for RT.

It is important, therefore, that the figure of the dentist be regularly involved in multidisciplinary TBs for the management of head and neck patients to improve patient quality of life as much as possible and to reduce the risk of complications following oncologic treatment.

## Figures and Tables

**Table 1:** General characteristics of the population and correlation with OH.

		Total Sample	Good OH	Poor OH	Significance
Gender	Men	128 (65.6%)	26	102	χ2 Test—*p* < 0.05
	Women	67 (34.4%)	23	44	
Age	Mean (range; SD)	60.4 (22–92; 12.4)	50.4 (22–86; 13.4)	63.7 (40–92; 10)	Pearson’s Correlation Analysis—*p* < 0.05
	<40	9 (4.6%)	9	0	
	40–49	26 (13.4%)	14	12	
	50–59	50 (25.6%)	13	37	
	60–69	65 (33.3%)	11	54	
	70–79	37 (19.0%)	0	37	
	>80	8 (4.1%)	2	6	
Tumor Type	SCC	173 (88.7%)	44	129	-
	Other types	22 (11.3%)	5	17	
Tumor Stage	Stage I	13 (6.7%)	3	10	-
	Stage II	31 (15.9%)	12	19	
	Stage III	49 (25.1%)	9	40	
	Stage IV	102 (52.3%)	25	77	
Tumor Site	Hypopharynx	6 (3.1%)	2	4	χ2 Test—*p* < 0.05
	Larynx	44 (22.6%)	6	38	
	Oral cavity	41 (21%)	9	32	
	Oropharynx	49 (25.1%)	13	36	
	Rhinopharynx	23 (11.8%)	13	10	
	Salivary glands	15 (7.7%)	2	13	
	Other sites	17 (8.7%)	4	13	
Smoking	Smokers	125 (64.1%)	22	103	χ2 Test—*p* < 0.05
	No smokers	70 (35.9%)	27	43	
Diabetes	Yes	11 (5.6%)	2	9	-
	No	184 (94.4%)	47	137	
Surgery ^a^	Performed	86 (44.1%)	17	69	-
	Not performed	109 (55.9%)	32	77	
Chemotherapy	Scheduled	104 (53.3%)	30	74	-
	Not scheduled	91 (46.7%)	19	72	
	Total	195 (100%)	49	146	

^a^ Major oncologic surgery (i.e., fibula free flap for mandible reconstruction, glossectomy). SCC: squamous cell carcinoma.

**Table 2:** Oral health parameters of the studied population and correlation with OH. SD: standard deviation.

		Total	Good OH	Poor OH	Significance
Edentulism	Edentulous patients	8 (4.1%)	0	8	-
	Non-edentulous patients	187 (95.9%)	49	138	
		195 (100%)			
Periodontitis	Affected patients	150 (76.9%)	16	134	χ2 Test—*p* < 0.05
	Non-affected patients	45 (23.1%)	33	12	
		195 (100%)			
Periodontal Staging	Stage I	21 (14%)	9	12	χ2 Test—*p* < 0.05
	Stage II	22 (14.7%)	7	15	
	Stage III	42 (28%)	0	42	
	Stage IV	65 (43.3%)	0	65	
		150 (100%)			
Periodontal Grading	Grade A	40 (26.7%)	5	35	-
	Grade B	66 (44%)	10	56	
	Grade C	44 (29.3%)	4	40	
		150 (100%)			
DMFt	Median (range)	16 (0–32)	8 (0–13)	20 (0–32)	Pearson’s Correlation Analysis—*p* < 0.05
DMFt ≥ 13	No	80 (41.0%)	49	31	χ2 Test—*p* < 0.05
	Yes	115 (59.0%)	0	115	
		195 (100%)			
Mouth Opening	Mean (range; SD)	38.8 (12–63; 10.1)	38.6 (12–54; 11.1)	39.1 (12–63; 9.8)	-
	<20 mm	21 (10.8%)	7	14	-
	≥20 mm	174 (89.2%)	42	132	
		195 (100%)			
		195 (100%)	49	146	

**Table 3:** General and oral health characteristics of the studied population, according to the localization of the tumor.

		Larynx 44	Oral Cavity 41	Oropharynx 49	Rhinopharynx 23	Salivary Glands 15	Other Sites 23	Total Sample 195	Significance
Gender	Male	35 (27.3%)	23 (18%)	38 (29.7%)	11 (8.6%)	9 (7%)	12 (9.4%)	128 (100%)	χ2 Test—*p* < 0.05
	Female	9 (13.4%)	18 (26.9%)	11 (16.4%)	12 (17.9%)	6 (9%)	11 (16.4%)	67 (100%)	
Age	<40	0 (0%)	2 (22.2%)	0 (0%)	4 (44.4%)	1 (11.1%)	2 (22.2%)	9 (100%)	Pearson’s Correlation Analysis—*p* < 0.05
	40–49	6 (23.1%)	5 (19.2%)	6 (23.1%)	4 (15.4%)	1 (3.8%)	4 (15.4%)	26 (100%)	
	50–59	12 (24%)	11 (22%)	14 (28%)	4 (8%)	4 (8%)	5 (10%)	50 (100%)	
	60–69	15 (23.1%)	12 (18.5%)	21 (32.3%)	9 (13.8%)	2 (3%)	6 (9.3%)	65 (100%)	
	70–79	11 (29.7%)	7 (18.9%)	8 (21.6%)	2 (5.4%)	4 (10.9%)	5 (13.5%)	37 (100%)	
	>80	0 (0%)	4 (50%)	0 (0%)	0 (0%)	3 (37.5%)	1 (12.5%)	8 (100%)	
Tumor Type	SCC	44 (25.4%)	39 (22.5%)	47 (27.1%)	22 (12.7%)	3 (1.7%)	18 (10.4%)	173 (100%)	-
	Other types	0 (0%)	2 (9.1%)	2 (9.1%)	1 (4.5%)	12 (54.4%)	5 (22.7%)	22 (100%)	
Tumor Stage	Stage I	3 (23%)	1 (7.7%)	4 (30.8%)	1 (7.7%)	2 (15.4%)	2 (15.4%)	13 (100%)	-
	Stage II	9 (29%)	3 (9.7%)	6 (19.4%)	6 (19.4%)	6 (19.4%)	1 (3.2%)	31 (100%)	
	Stage III	9 (18.4%)	8 (16.3%)	16 (32.6%)	7 (14.4%)	3 (6.1%)	6 (12.2%)	49 (100%)	
	Stage IV	23 (22.6%)	29 (28.4%)	23 (22.6%)	9 (8.8%)	4 (3.9%)	14 (13.7%)	102 (100%)	
Chemotherapy	Scheduled	15 (14.4%)	18 (17.4%)	38 (36.5%)	17 (16.3%)	4 (3.8%)	12 (11.6%)	104 (100%)	χ2 Test—*p* < 0.05
	Not scheduled	29 (31.9%)	23 (25.4%)	11 (12%)	6 (6.7%)	11 (12%)	11 (12%)	91 (100%)	
Surgery	Performed	19 (22.1%)	35 (40.7%)	6 (7%)	3 (3.5%)	13 (15.1%)	10 (11.6%)	86 (100%)	χ2 Test—*p* < 0.05
	Not performed	25 (23%)	6 (5.5%)	43 (39.4%)	20 (18.3%)	2 (1.9%)	13 (11.9%)	109 (100%)	
Smoking	Yes	38 (30.4%)	25 (20%)	34 (27.2%)	10 (8%)	6 (4.8%)	12 (9.6%)	125 (100%)	χ2 Test—*p* < 0.05
	No	6 (8.6%)	16 (22.9%)	15 (21.4%)	13 (18.6%)	9 (12.9%)	11 (15.6%)	70 (100%)	
Edentulism	Yes	3 (37.5%)	2 (25%)	1 (12.5%)	0 (0%)	2 (25%)	0 (0%)	8 (100%)	-
	No	41 (22%)	39 (20.8%)	48 (25.7%)	23 (12.3%)	13 (6.9%)	23 (12.3%)	187 (100%)	
Periodontitis	Not affected	6 (13.3%)	10 (22.2%)	9 (20%)	11 (24.5%)	3 (6.7%)	6 (13.3%)	45 (100%)	-
	Stage I and II	8 (18.7%)	6 (13.9%)	15 (34.9%)	5 (11.7%)	3 (6.9%)	6 (13.9%)	43 (100%)	
	Stage III and IV	30 (28%)	25 (23.4%)	25 (23.4%)	7 (6.5%)	9 (8.4%)	11 (10.3%)	107 (100%)	
DMFt	Median	21	15	16	10	19	14	16	-
DMFt ≥ 13	No	11 (13.8%)	18 (22.5%)	22 (27.5%)	15 (18.8%)	3 (3.7%)	11 (13.7%)	80 (100%)	χ2 Test—*p* < 0.05
	Yes	33 (28.7%)	23 (20%)	27 (23.5%)	8 (7%)	12 (10.4%)	12 (10.4%)	115 (100%)	
Mouth Opening (mm)	<25	3 (14.3%)	7 (33.3%)	6 (28.6%)	1 (4.8%)	2 (9.5%)	2 (9.5%)	21 (100%)	-
	≥25	41 (23.6%)	34 (19.5%)	43 (24.7%)	22 (12.6%)	13 (7.5%)	21 (12.1%)	174 (100%)	
Oral Health	Good	6 (12.2%)	9 (18.5%)	13 (26.5%)	13 (26.5%)	2 (4.1%)	6 (12.2%)	49 (100%)	χ2 Test—*p* < 0.05
	Poor	38 (26%)	32 (21.9%)	36 (24.7%)	10 (6.8%)	13 (8.9%)	17 (11.7%)	146 (100%)	

**Table 4:** General and oral health characteristics of the studied population, according to the habit of smoking.

		Smokers 125	Non-Smokers 70	Total Sample 195	Significance
Gender	Male	92 (71.9%)	36 (28.1%)	128 (100%)	χ2 Test—*p* < 0.05
	Female	33 (49.2%)	34 (50.8%)	67 (100%)	
Age	<40	4 (44.4%)	5 (55.6%)	9 (100%)	-
	40–49	16 (61.5%)	10 (38.5%)	26 (100%)	
	50–59	34 (68%)	16 (32%)	50 (100%)	
	60–69	43 (66.2%)	22 (33.8%)	65 (100%)	
	70–79	24 (64.9%)	13 (35.1%)	37 (100%)	
	>80	4 (50%)	4 (50%)	8 (100%)	
Tumor Type	SCC	115 (66.5%)	58 (33.5%)	173 (100%)	
	Other types	10 (45.6%)	12 (54.5%)	22 (100%)	
Tumor Site	Larynx	38 (86.3%)	6 (13.7%)	44 (100%)	χ2 Test—*p* < 0.05
	Oral cavity	25 (61%)	16 (39%)	41 (100%)	
	Oropharynx	34 (69.4%)	15 (30.6%)	49 (100%)	
	Rhinopharynx	10 (43.5%)	13 (56.5%)	23 (100%)	
	Salivary glands	6 (40%)	9 (60%)	15 (100%)	
	Other sites	12 (52.2%)	11 (47.8%)	23 (100%)	
Tumor Stage	Stage I	7 (53.8%)	6 (46.2%)	13 (100%)	
	Stage II	15 (48.4%)	16 (51.6%)	31 (100%)	
	Stage III	29 (59.2%)	20 (40.8%)	49 (100%)	
	Stage IV	74 (72.6%)	28 (27.4%)	102 (100%)	
Chemotherapy	Scheduled	68 (65.4%)	36 (34.6%)	104 (100%)	
	Not scheduled	57 (62.6%)	34 (37.4%)	91 (100%)	
Surgery	Performed	52 (60.5%)	34 (39.5%)	86 (100%)	
	Not performed	73 (67%)	36 (33%)	109 (100%)	
Edentulism	Yes	4 (50%)	4 (50%)	8 (100%)	
	No	121 (64.7%)	66 (35.3%)	187 (100%)	
Periodontitis	Not affected	19 (42.2%)	26 (57.8%)	45 (100%)	χ2 Test—*p* < 0.05
	Stage I and II	26 (60.5%)	17 (39.5%)	43 (100%)	
	Stage III and IV	80 (74.8%)	27 (25.2%)	107 (100%)	
DMFt	Mean	17.8	15.3	16.9	
	Median	18	13	16	
DMFt ≥ 13	No	43 (53.8%)	37 (46.2%)	80 (100%)	χ2 Test—*p* < 0.05
	Yes	82 (71.3%)	33 (28.7%)	115 (100%)	
Mouth Opening (mm)	<25	14 (66.6%)	7 (33.4%)	21 (100%)	
	≥25	111 (63.8%)	63 (36.2%)	174 (100%)	
Oral Health	Good	22 (44.9%)	27 (55.1%)	49 (100%)	χ2 Test—*p* < 0.05
	Poor	103 (70.5%)	43 (29.5%)	146 (100%)	

**Table 5:** General and oral health characteristics of the studied population, according to the age of the subjects (decades).

		Age of the Subjects						Total Sample	Significance
		<40 9 (4.6%)	40–49 26 (13.3%)	50–59 50 (25.7%)	60–69 65 (33.3%)	70–79 37 (19%)	>80 8 (4.1%)	195 (100%)	
Gender	Male	4 (3.1%)	17 (13.3%)	30 (23.4%)	52 (40.6%)	21 (16.4%)	4 (3.1%)	128 (100%)	
	Female	5 (7.5%)	9 (13.4%)	20 (29.9%)	13 (19.4%)	16 (23.8%)	4 (6%)	67 (100%)	
Tumor Type	SCC	6 (3.5%)	25 (14.5%)	44 (25.3%)	60 (34.7%)	32 (18.5%)	6 (3.5%)	173 (100%)	
	Other types	3 (13.7%)	1 (4.5%)	6 (27.3%)	5 (22.7%)	5 (22.7%)	2 (9.1%)	22 (100%)	
Tumor Site	Larynx	0 (0%)	6 (13.7%)	12 (27.2%)	15 (34.1%)	11 (25%)	0 (0%)	44 (100%)	χ2 Test—*p* < 0.05
	Oral cavity	2 (4.9%)	5 (12.2%)	11 (26.8%)	12 (29.4%)	7 (17%)	4 (9.7%)	41 (100%)	
	Oropharynx	0 (0%)	6 (12.2%)	14 (28.6%)	21 (42.9%)	8 (16.3%)	0 (0%)	49 (100%)	
	Rhinopharynx	4 (17.4%)	4 (17.4%)	4 (17.4%)	9 (39.1%)	2 (8.7%)	0 (0%)	23 (100%)	
	Salivary glands	1 (6.7%)	1 (6.6%)	4 (26.7%)	2 (13.3%)	4 (26.7%)	3 (20%)	15 (100%)	
	Other sites	2 (8.7%)	4 (17.4%)	5 (21.7%)	6 (26.9%)	5 (21.7%)	1 (4.3%)	23 (100%)	
Tumor Stage	Stage I	0 (0%)	3 (23.1%)	4 (30.8%)	2 (15.3%)	4 (30.8%)	0 (0%)	13 (100%)	
	Stage II	2 (6.5%)	3 (9.7%)	8 (25.8%)	10 (32.3%)	6 (19.3%)	2 (6.4%)	31 (100%)	
	Stage III	1 (2%)	6 (12.2%)	14 (28.6%)	19 (38.8%)	8 (16.3%)	1 (2%)	49 (100%)	
	Stage IV	6 (5.9%)	14 (13.7%)	24 (23.6%)	34 (33.3%)	19 (18.6%)	5 (4.9%)	102 (100%)	
Chemotherapy	Scheduled	5 (4.8%)	17 (16.4%)	36 (34.6%)	35 (33.7%)	10 (9.6%)	1 (0.9%)	104 (100%)	χ2 Test—*p* < 0.05
	Not scheduled	4 (4.4%)	9 (9.9%)	14 (15.4%)	30 (32.9%)	27 (29.7%)	7 (7.7%)	91 (100%)	
Surgery	Performed	2 (2.4%)	12 (13.9%)	24 (27.9%)	21 (24.4%)	22 (25.6%)	5 (5.8%)	86 (100%)	
	Not performed	7 (6.4%)	14 (12.8%)	26 (23.9%)	44 (40.3%)	15 (13.8%)	3 (2.8%)	109 (100%)	
Smoking	Yes	4 (3.2%)	16 (12.8%)	34 (27.2%)	43 (34.4%)	24 (19.2%)	4 (3.2%)	125 (100%)	
	No	5 (7.1%)	10 (14.3%)	16 (22.9%)	22 (31.4%)	13 (18.6%)	4 (5.7%)	70 (100%)	
Edentulism	Yes	0 (0%)	0 (0%)	0 (0%)	2 (25%)	5 (62.5%)	1 (12.5%)	8 (100%)	χ2 Test—*p* < 0.05
	No	9 (4.8%)	26 (13.9%)	50 (26.7%)	63 (33.7%)	32 (17.1%)	7 (3.7%)	187 (100%)	
Periodontitis	Not affected	7 (15.6%)	12 (26.7%)	15 (33.3%)	8 (17.9%)	2 (4.4%)	1 (2.2%)	45 (100%)	χ2 Test—*p* < 0.05
	Stage I and II	2 (4.7%)	7 (16.4%)	13 (30.2%)	17 (39.5%)	2 (4.6%)	2 (4.6%)	43 (100%)	
	Stage III and IV	0 (0%)	7 (6.5%)	23 (21.5%)	40 (37.4%)	32 (29.9%)	5 (4.7%)	107 (100%)	
DMFt	Median	5	8	16	16	25	23	16	Pearson’s Correlation Analysis—*p* < 0.05
DMFt ≥ 13	No	9 (11.3%)	19 (23.8%)	17 (21.2%)	28 (35%)	5 (6.2%)	2 (2.5%)	80 (100%)	χ2 Test—*p* < 0.05
	Yes	0 (0%)	7 (6.1%)	33 (28.7%)	37 (32.2%)	32 (27.9%)	6 (5.2%)	115 (100%)	
Mouth Opening (mm)	<25	2 (9.5%)	3 (14.3%)	9 (42.8%)	4 (19%)	3 (14.3%)	0 (0%)	21 (100%)	-
	≥25	7 (4%)	23 (13.3%)	41 (23.7%)	61 (35%)	34 (19.5%)	8 (4.6%)	174 (100%)	
Oral Health	Good	9 (18.4%)	14 (28.6%)	13 (26.5%)	11 (22.3%)	0 (0%)	2 (4.1%)	49 (100%)	χ2 Test—*p* < 0.05
	Poor	0 (0%)	12 (8.2%)	37 (25.4%)	54 (36.9%)	37 (25.4%)	6 (4.1%)	146 (100%)	

## Data Availability

The data presented in this study are available on request from the corresponding author. The data are not publicly available due to privacy.

## Supplementary Materials

The following supporting information can be downloaded at: https://www.mdpi.com/article/10.3390/cancers14061411/s1, Table S1: STROBE Statement—Checklist of items that should be included in reports of ***cohort studies***.
